# Molecular pathways, tissue-specific roles, and therapeutic opportunities in mitochondrial resilience-based strategies for insulin resistance

**DOI:** 10.3389/fcdhc.2026.1790182

**Published:** 2026-04-29

**Authors:** Yousef M. Almoghrabi, Basmah M. Eldakhakhny, Ghada Ajabnoor, Faisal Alandejani, Ahmad Jamal, Dareen Alyousfi, Ahmad M. Alzahrani, Mostafa A. Elsamanoudy, Taghreed Shamrani, Ayman Z. Elsamanoudy

**Affiliations:** 1 Clinical Biochemistry Department, Faculty of Medicine, King Abdulaziz University, Jeddah, Saudi Arabia; 2 Regenerative Medicine Unit, King Fahd Medical Research Center, King Abdulaziz University, Jeddah, Saudi Arabia; 3 Food, Nutrition, and Lifestyle Research Unit, King Fahd Medical Research Centre, King Abdulaziz University, Jeddah, Saudi Arabia; 4 Faculty of Medicine, Horus University, New Damietta, Damietta, Egypt; 5 Medical Biochemistry and Molecular Biology Department, Faculty of Medicine, Mansoura University, Mansoura, Egypt

**Keywords:** insulin resistance, lifestyle interventions, metabolic health, mitochondrial biogenesis, mitochondrial dysfunction, mitochondrial resilience, mitophagy, oxidative stress

## Abstract

Insulin resistance (IR) is characterized by impaired insulin signaling in skeletal muscle, liver, and adipose tissue. Increasing evidence indicates mitochondrial dysfunction as a key factor contributing to IR. Mitochondrial resilience refers to the mitochondria’s ability to adapt to metabolic stress by regulating biogenesis, dynamics, mitophagy, and redox homeostasis. Linking mitochondrial resilience with insulin signaling could be essential for maintaining metabolic health. This review aims to map and synthesize the existing literature on the relationship between mitochondrial resilience and insulin resistance, focusing on cellular and molecular mechanisms, tissue-specific roles, metabolic consequences, and translational evidence from both animal and human studies. This scoping review adhered to PRISMA-ScR guidelines and included comprehensive searches of PubMed, Scopus, Web of Science, Embase, and Google Scholar. It included experimental and observational studies, original articles, systematic reviews, and meta-analyses, while excluding non-English publications and animal studies without clinical relevance. A total of 7, 012 records were identified; after removing duplicates, screening, and assessing eligibility, 184 studies were included. The evidence shows that impaired mitochondrial biogenesis, defective mitochondrial dynamics, reduced mitophagy, and oxidative stress disturb insulin signaling and promote metabolic inflexibility. Conversely, enhancing mitochondrial resilience increases mitochondrial quantity and function. Lifestyle modification strategies and pharmacological intervention target these pathways to improve mitochondrial resilience. Importantly, understanding inter-individual differences in mitochondrial adaptive capacity may support the development of personalized therapeutic and nutritional strategies aimed at improving insulin sensitivity and metabolic outcomes. In conclusion, mitochondrial resilience provides a comprehensive framework connecting mitochondrial quality to insulin signaling and metabolic health. Focusing on mitochondrial resilience is a promising, mechanism-based strategy for preventing and managing insulin resistance and its related comorbidities.

## Introduction

Insulin resistance (IR) arises when cells fail to adequately respond to insulin, leading to challenges in insulin-dependent glucose uptake in the skeletal muscles, liver, and adipose tissue (1). IR is associated with hyperinsulinemia and impaired glucose tolerance (2). The development of IR is influenced by the interplay of various factors, including genetic factors, excess central body fat accumulation, low-grade inflammation, and disturbed mitochondrial function. These factors result in the emergence of this metabolic condition (3).

Mitochondrial dysfunction represents a pivotal factor in the emergence of insulin resistance, which is a hallmark feature of type 2 diabetes and metabolic syndrome (4, 5). When mitochondrial functionality is impaired, these organelles fail to efficiently oxidize substrates, leading to an augmented generation of reactive oxygen species (ROS). Such ROS can potentially damage cellular components and provoke proinflammatory mechanisms, finally adversely affecting insulin sensitivity (6, 7).

Mitochondria are indispensable for the metabolism of lipids and glucose, both of which are critical for energy synthesis. Disruptions within these metabolic pathways result in diminished ATP production and compromised oxidative phosphorylation, processes that are essential for maintaining insulin sensitivity (8).

Genetic variations that affect mitochondrial biogenesis may play a role in developing insulin resistance within target tissues. Enhancing mitochondrial biogenesis may improve insulin sensitivity and help prevent metabolic disorders by reducing mitochondrial oxidative stress through agents such as mitoquinone, thereby facilitating insulin-mediated glucose uptake in skeletal muscle (Matteo Fiorenza et al., 2024). This understanding suggests a promising therapeutic strategy for improving insulin sensitivity by enhancing mitochondrial integrity (9).

Modifications in lifestyle, including increased physical activity and caloric restriction, can reinforce mitochondrial performance (10, 11). These modifications promote metabolic equilibrium and enhance insulin sensitivity by adopting thermogenesis and the browning of white adipose tissue (12, 13).

Mitochondrial resilience is a crucial aspect of cellular health, referring to the capacity of mitochondria to adapt to various stressors, maintain homeostasis, and prevent dysfunction. This resilience encompasses several key processes, including mitochondrial dynamics, specifically fusion and fission, biogenesis, and mitophagy. These processes are vital for sustaining cellular energy metabolism, significantly preventing metabolic disorders (14). The importance of mitochondrial resilience is underscored by its involvement in various pathophysiological mechanisms, particularly in insulin resistance.

The term mitochondrial resilience refers to the capacity of mitochondria to maintain or restore functional integrity when exposed to metabolic, oxidative, or environmental stress. This adaptive capability arises from the coordinated regulation of key mitochondrial processes, including mitochondrial biogenesis, mitochondrial dynamics (fusion and fission), mitophagy, and redox homeostasis. Through these mechanisms, cells are able to preserve mitochondrial quality, sustain ATP production, and maintain metabolic flexibility under conditions that challenge cellular energy balance. Such resilience is essential for cellular homeostasis and is particularly important in high-energy-demand organs such as the heart and brain (15). Understanding the mechanisms that support mitochondrial resilience may therefore provide important insights into therapeutic strategies for diseases associated with mitochondrial dysfunction.

Mitochondrial resilience is closely related to, but conceptually distinct from, other commonly used terms in mitochondrial biology. Mitochondrial quality control refers to the molecular surveillance systems that detect, repair, or eliminate damaged mitochondrial components to maintain cellular energy metabolism (15). Similarly, mitochondrial plasticity describes the ability of mitochondria to dynamically adjust their structure, abundance, and metabolic activity in response to physiological demands (16). While these mechanisms contribute to mitochondrial adaptation, mitochondrial resilience represents a broader integrative framework that encompasses these processes and emphasizes the overall capacity of mitochondria to withstand and recover from metabolic stress (17). Related processes such as mitochondrial dynamics and quality control are therefore considered key contributors to this adaptive capacity (18).

Within this framework, mitochondrial resilience can be viewed both as a biological adaptive trait, reflecting the intrinsic capacity of cells to cope with metabolic challenges, and as a potential therapeutic target for interventions aimed at restoring mitochondrial function in metabolic disorders such as insulin resistance (19). Enhancing mitochondrial resilience may improve insulin sensitivity and overall metabolic health, highlighting its potential as a promising focus for future research and therapeutic development (20).

Despite growing recognition of the role of mitochondrial dysfunction in insulin resistance (IR), there is a lack of comprehensive understanding of how mitochondrial resilience modulates IR development and progression, as the published reviews often isolate specific pathways or interventions without integrating mechanistic insights with translational evidence. There is also insufficient clarity regarding how mitochondrial quality control relates to IR in different tissues. Moreover, the potential of lifestyle and nonpharmacological interventions to restore or enhance mitochondrial Resilience in metabolic dysfunction remains underexplored in an integrated framework.

This scoping review aims to map the existing literature on the relationship between mitochondrial resilience and insulin resistance by examining the cellular and molecular mechanisms that connect mitochondrial function to insulin signaling and exploring the tissue-specific roles of mitochondrial resilience in modulating insulin sensitivity. It will also assess lifestyle, dietary, and nonpharmacological interventions targeting mitochondrial function to improve metabolic outcomes. Both animal and human studies will be included to provide a translational perspective, and the review will highlight evidence gaps and inconsistencies across studies, proposing directions for future research.

The objectives of this scoping review are to identify and categorize the molecular and cellular mechanisms through which mitochondrial resilience influences insulin signaling. The second objective is to examine the tissue-specific roles of mitochondrial dysfunction in the development of insulin resistance. Moreover, it aims to assess the clinical and metabolic consequences of compromised mitochondrial function in insulin-resistant conditions, to review current dietary, lifestyle, and pharmacological interventions that support mitochondrial health, and to evaluate their effectiveness in improving insulin sensitivity. Additionally, the review outlines future directions for targeted investigations in the context of mitochondrial resilience and metabolic disorders.

## Methods

**Figure 1:**
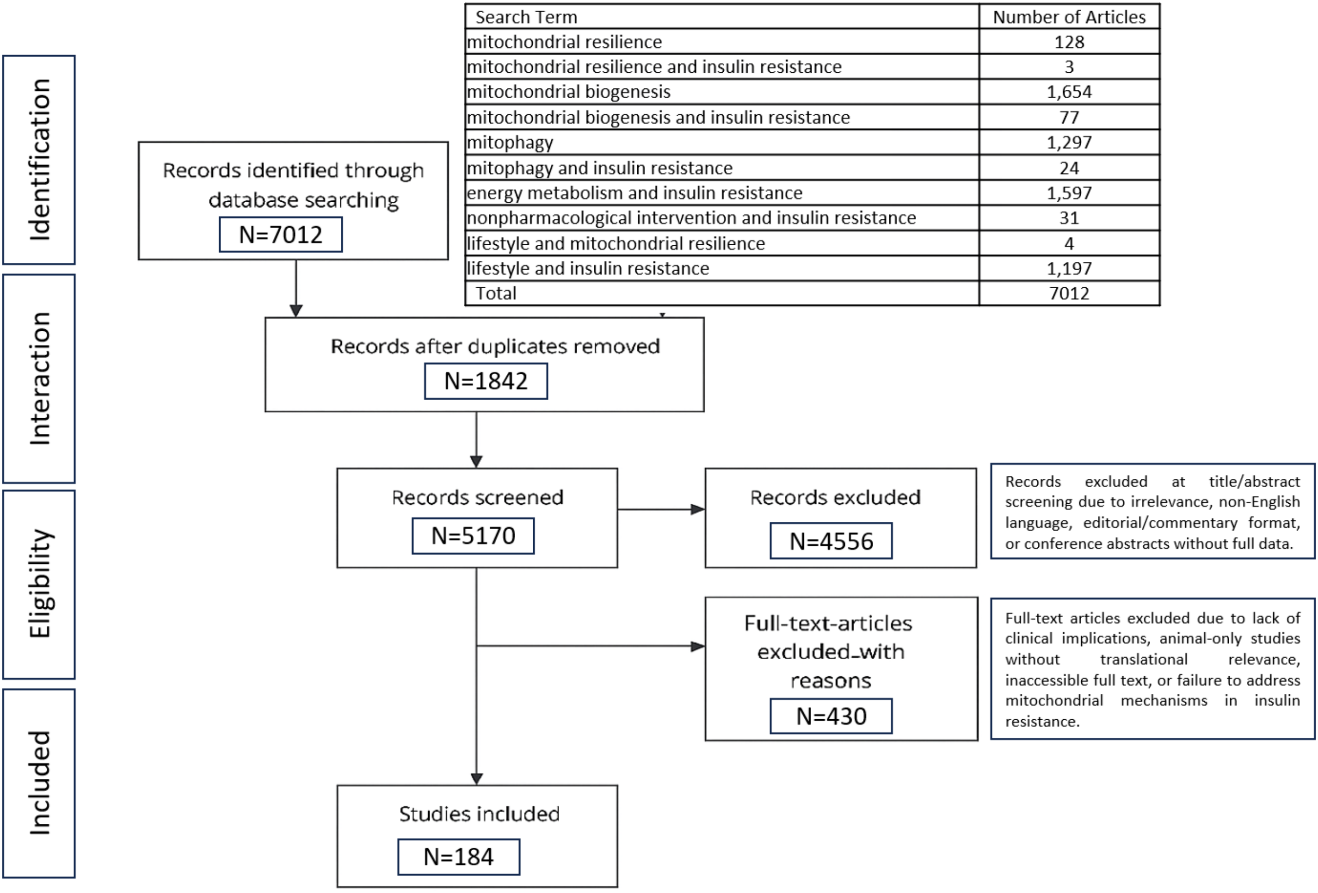
PRISMA flow diagram of study selection. The diagram summarizes the study selection process following PRISMA-ScR guidelines. A total of 7, 012 records were identified through database searching. After removing 1, 842 duplicates, 5, 170 records were screened, and 4, 556 were excluded at the title and abstract level. 614 full-text articles were assessed for eligibility, of which 430 were excluded for lack of clinical relevance, inadequate translational value, inaccessible full text, or failure to address mitochondrial mechanisms in insulin resistance. Ultimately, 184 studies were included in the scoping review.

In this scoping review, the authors conducted a detailed literature search using databases such as PubMed, Scopus, Web of Science, and Google Scholar to achieve a comprehensive overview. This scoping review was conducted in accordance with the PRISMA-ScR (Preferred Reporting Items for Systematic Reviews and Meta-Analyses Extension for Scoping Reviews) guidelines to ensure methodological rigor and transparency (**Figure 1**).

This review included studies investigating the relationship between mitochondrial function or resilience and insulin resistance, focusing on mechanistic pathways, tissue-specific effects, metabolic outcomes, or interventions to enhance mitochondrial health. Eligible studies were experimental or observational, involving human participants and animal models with clinical implications, including original research, systematic reviews, or meta-analyses. Articles were required to be published in English and to have accessible full texts. Studies were excluded if they were conference abstracts without full data, editorials, commentaries, non-English publications, or did not address mitochondrial function in insulin resistance. Animal studies without clinical implications were also excluded.

A comprehensive literature search was conducted in PubMed, Scopus, Web of Science, and Embase databases from inception to the most recent date. Keywords and MeSH terms were based on combinations of the following: “mitochondrial resilience”, “mitochondrial dysfunction”, “insulin resistance”, “mitochondrial biogenesis”, “oxidative stress”, “mitophagy”, “energy metabolism”, “intervention”, “exercise”, “lifestyle”, and “nonpharmacological intervention”.

The article selection process was conducted in two sequential stages. First, titles and abstracts were screened to exclude studies that did not meet the eligibility criteria. Subsequently, the full texts of the remaining articles were carefully reviewed to determine their relevance according to the predefined inclusion criteria.

Data extraction was performed independently by two authors, and any discrepancies were resolved through discussion and consensus. A previously prepared data extraction form was used to collect relevant information from the included studies, covering study characteristics (including author, year, design, and population or model), types and measurements of mitochondrial parameters, mechanistic insights into insulin signaling, reported clinical, metabolic, and molecular outcomes, interventions applied and their effects on mitochondrial and insulin function, as well as the studies’ limitations and identified research gaps. The extracted data were synthesized through descriptive and thematic analysis, with findings categorized based on mechanisms, intervention strategies, and associated health outcomes to identify existing patterns and gaps in the literature. A narrative synthesis, supported by visual mapping where appropriate, was conducted to summarize current knowledge and identify areas requiring further research.

As this review was based exclusively on previously published literature and did not include the use of identifiable patient data, ethical approval was not required according to institutional regulations.

### The roles of mitochondria in energy production and metabolic regulation

Mitochondria play a vital role in energy production through oxidative phosphorylation. This complex process occurs within the inner mitochondrial membrane, where a series of protein complexes known as the electron transport chain facilitates the transfer of electrons derived from nutrients (21). Consequently, mitochondria are essential for converting the chemical energy found in food into a usable form of energy crucial for various cellular activities (22).

Besides their energy-generating function, mitochondria play a significant role in metabolic regulation. They are involved in various metabolic pathways, including the citric acid cycle (Krebs cycle) (23). Mitochondria also participate in fatty acid metabolism through β-oxidation. Additionally, they help balance energy production and consumption, ensuring that cells can effectively adjust to fluctuating energy demands, such as during exercise or fasting (24).

Furthermore, mitochondria are vital for cellular signaling and homeostasis. They generate reactive oxygen species (ROS) as byproducts of ATP production, which can serve as signaling molecules affecting various cellular functions, including apoptosis and inflammation (25). Moreover, mitochondria are involved in the storage and regulation of calcium, which is critical for cellular signaling pathways (26). A detailed description of the mechanism, metabolic impact, as well as the health effects of the different roles of mitochondria in energy production and metabolic regulation are listed in **Table 1**.

**Table 1:** The mechanism, metabolic impact, and health effects of the role of mitochondria in energy production and metabolic regulation.

Role of mitochondria	Mechanism	Metabolic impact	Health effect	Reference
ATP production via oxidative phosphorylation	Electron transport chain (ETC) and chemiosmosis in the inner mitochondrial membrane (27, 28)	Efficient generation of ATP from glucose and fatty acids (29)	Supports cellular energy demands. Dysfunction leads to fatigue and organ failure (30, 31)	(27–31).
Regulation of glucose metabolism	Pyruvate oxidation in the TCA cycle and NADH/FADH_2_ production	Links glycolysis with oxidative phosphorylation metabolism (32)	Mitochondrial defects may impair glucose utilization, contributing to insulin resistance (33).	(25, 26)
Fatty acid(FA) β-oxidation	Breakdown of long-chain fatty acids in the mitochondrial matrix	Provides acetyl-CoA for the TCA cycle and ketone body production	Impaired FA oxidation contributes to the development of adiposity and metabolic syndrome	(34, 35)
Amino acid metabolism	Transamination, deamination, and the urea cycle (in liver mitochondria)	Supplies intermediates for gluconeogenesis and the TCA cycle	Disruptions affect nitrogen balance and blood glucose homeostasis regulation	(36)
Reactive oxygen species (ROS) regulation	ETC leakage of electrons and antioxidant defenses (e.g., SOD, glutathione) (37)	Maintains redox balance; ROS act as signaling molecules	Excessive ROS causes oxidative stress, inflammation, and aging-related diseases (38, 39)	(37–39)
Apoptosis regulation	Release of cytochrome c and activation of the caspase cascade (40)	Controls cell turnover and prevents damaged cell survival	Dysfunction linked to cancer, neurodegeneration, and autoimmune disorders.	(40, 41)
Calcium homeostasis	Mitochondrial calcium uniporter and buffering capacity (42)	Regulates metabolism and signaling pathways (43)	Dysregulation is linked to insulin resistance, cardiac dysfunction, and neurotoxicity. (44, 45)	(42–45)
Thermogenesis (in brown fat and skeletal muscle)	Uncoupling protein 1 (UCP1) activity uncouples respiration from ATP synthesis (46). Mitochondria regulate ATP production and fatty acid β-oxidation in myocytes; coordinated by PGC-1α-driven biogenesis and mitochondrial dynamics (fusion/fission) to meet energy demand through overexpression of UCP3 (47)	Drive away energy as heat	Impacts body temperature regulation and energy expenditure. Dysregulation leads to the development of adiposity (48)	(46–48)
Mitochondrial biogenesis and turnover	Regulated by PGC-1α, NRF1, TFAM, mitophagy processes	Maintains mitochondrial quality and adaptability to metabolic demands	Defects linked to aging, metabolic diseases, and reduced exercise capacity	(49, 50)

### Mitochondrial resilience and its importance in cellular health

Mitochondrial resilience refers to the ability of mitochondria to adapt, recover, and maintain their function under various stressors, such as oxidative stress and hypoxia. This concept is crucial for understanding mitochondrial health and its role in cellular homeostasis (25). Key mechanisms of mitochondrial resilience include Mitochondrial Quality Control (MQC), which involves mitochondrial biogenesis, dynamics (fusion and fission), and mitophagy to maintain mitochondrial integrity (51).

Factors like PGC-1α regulate mitochondrial biogenesis, while fusion and fission are controlled by proteins like MFN1/2 and DRP1, respectively (52). Mitophagy, regulated by PINK1 and Parkin, removes damaged mitochondria to prevent dysfunction. The Mitochondrial Unfolded Protein Response (mtUPR) also helps manage misfolded proteins and is linked to biogenesis and dynamics (53) Mitochondria monitor their functional status and activate stress responses to restore homeostasis. Defective mitochondrial resilience can lead to metabolic disorders, neurodegenerative diseases, and cancer (54–56). **Table 2** presents mitochondrial resilience’s main mechanisms and metabolic roles.

**Table 2:** Key mechanisms and metabolic roles in mitochondrial resilience.

Mechanism	Role in mitochondrial resilience	Reference
Mitochondrial Biogenesis	Synthesis of new mitochondrial components to replace damaged or aged mitochondria	(16, 51)
Mitochondrial Dynamics	Fusion and fission processes that maintain mitochondrial shape and function	(51, 57)
Mitophagy	The selective autophagy pathway that removes damaged or dysfunctional mitochondria	(53, 58)
mtUPR	Stress response pathway that restores mitochondrial proteostasis	(53, 57)

### Insulin resistance

Insulin Resistance (IR) is a pathological condition characterized by the impaired ability of cells to respond effectively to insulin (3). This impairment disrupts glucose uptake and utilization, leading to compensatory hyperinsulinemia and hyperglycemia (59). A growing body of evidence connects mitochondrial dysfunction to the development and progression of IR (60). When mitochondrial function is compromised due to excess nutrient load, oxidative stress, or impaired biogenesis, cells exhibit reduced energy efficiency, increased reactive oxygen species (ROS) production, and altered substrate metabolism, all of which contribute to insulin signaling disruption (61, 62).

Emerging research introduces the concept of mitochondrial resilience, defined as the organelle’s ability to maintain or restore function in response to metabolic stress. Resilient mitochondria can adapt through enhanced biogenesis, efficient turnover (via mitophagy), and optimized oxidative capacity, thus preserving cellular insulin sensitivity (63, 64). Consequently, strengthening mitochondrial resilience presents a promising therapeutic avenue for improving metabolic health and combating insulin resistance (65–67).

Mitochondrial dysfunction plays a significant role in the pathophysiology of insulin resistance, a hallmark of T2DM. Impaired mitochondrial function can disrupt insulin signaling pathways, primarily through the production of reactive oxygen species (ROS) and inflammation, which are critical in the development of insulin resistance (68). This dysfunction affects various tissues, including adipose tissue, liver, and muscle, which are crucial for maintaining systemic insulin sensitivity. The following sections delve into these mechanisms and their implications (69). **Table 3** presents the link between the main mitochondrial function and the impact of insulin resistance.

**Table 3:** The interplay between mitochondrial function and insulin resistance implications.

Aspect	Mitochondrial function	Insulin resistance implications
Energy Production	Generates ATP through oxidative phosphorylation (70)	Impaired energy production contributes to metabolic dysregulation (71)
Metabolic Regulation	Regulates fatty acid oxidation and glucose metabolism (70, 72)	Dysregulation leads to hyperglycemia and hyperinsulinemia (73)
Resilience Mechanisms	Mitochondrial biogenesis, dynamics, and mitophagy (74)	Enhances insulin sensitivity and glucose metabolism (75, 76)
Disease Implications	Implicated in diabetes, neurodegeneration, and cardiovascular diseases (75)	Associated with type 2 diabetes, NAFLD, and cardiovascular diseases (59, 77, 78)

### Mitochondrial biogenesis and insulin sensitivity

**Figure 2:**
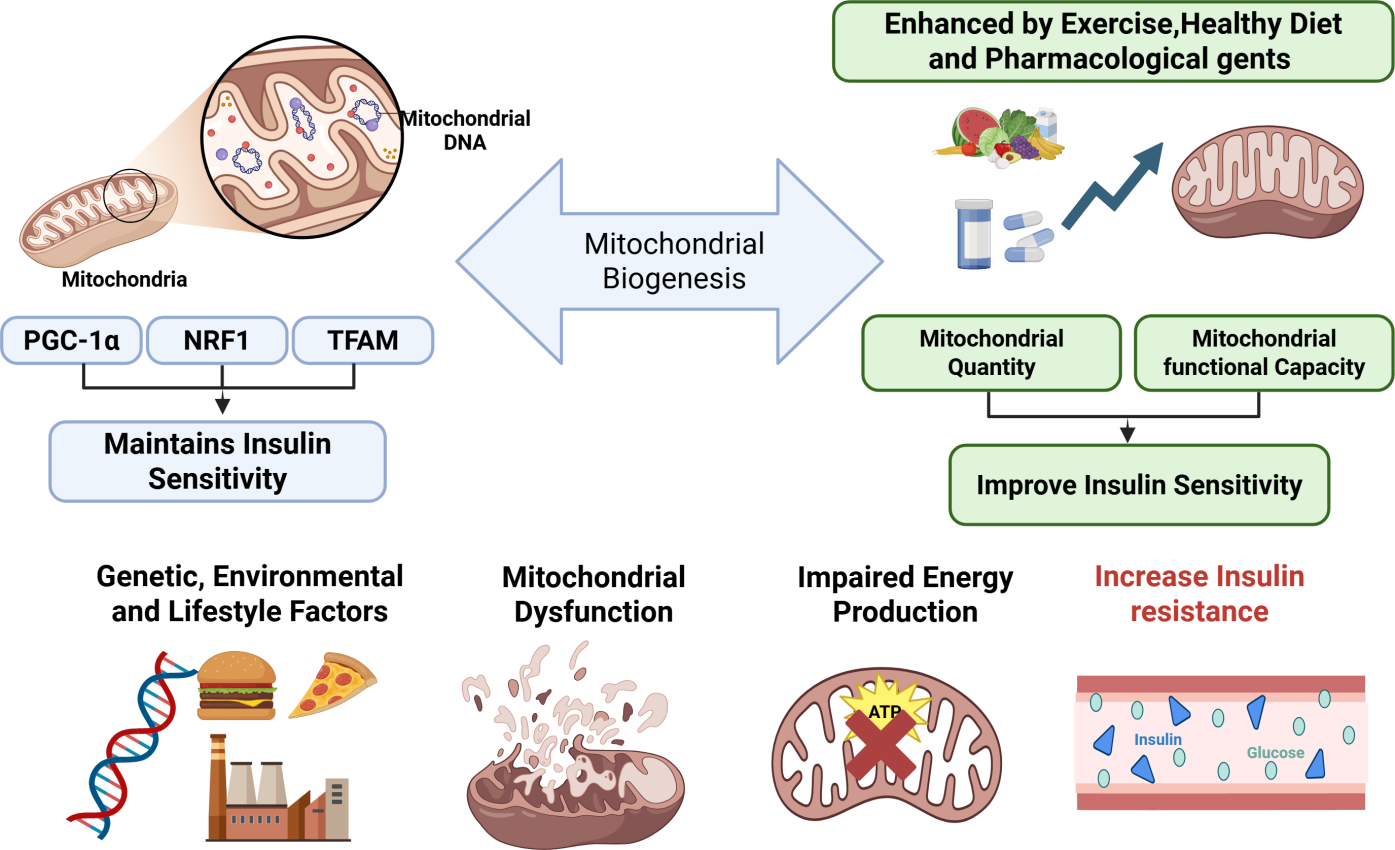
Mitochondrial biogenesis and insulin sensitivity. Regulation of mitochondrial biogenesis improves mitochondrial function and insulin sensitivity, while dysfunction promotes insulin resistance. Exercise, a healthy diet, and pharmacological interventions enhance this pathway within the multifactorial context of insulin resistance.PGC-1a, peroxisome proliferator-activated receptor γ co-activator 1α; NRF1, nuclear respiratory factor 1; TFAM, mitochondrial transcription factor A. The lower pathway shows how genetic susceptibility and adverse environmental or lifestyle factors, such as nutrient excess and physical inactivity, can lead to mitochondrial dysfunction. This results in impaired energy production and metabolic inflexibility, which disrupts insulin signaling and contributes to the development of insulin resistance. Together, these pathways illustrate how mitochondrial function critically influences metabolic health and insulin sensitivity. Created in BioRender. Eldakhakhny, B. (2026). https://BioRender.com/ofkcc8y.

Mitochondrial biogenesis is a complex process that is crucial for maintaining insulin sensitivity over time. It is regulated by essential factors such as the peroxisome proliferator-activated receptor γ co-activator 1α (PGC-1α), nuclear respiratory factor 1 (NRF1), and mitochondrial transcription factor A (TFAM) (4, 79). Enhancing mitochondrial biogenesis through targeted interventions may improve insulin sensitivity by increasing mitochondria’s quantity and functional capacity (66). This approach presents promising therapeutic options for individuals suffering from T2DM. While it is abundantly clear that mitochondrial dysfunction plays a pivotal and critical role in the complex landscape of insulin resistance, it remains essential to consider the multifactorial aspects that characterize T2DM as a whole (80). Additional elements, including various genetic factors, diverse environmental impacts, and myriad lifestyle decisions, also play integral roles in the intricate onset and advancement of insulin resistance, thus emphasizing the need for a holistic understanding (81). Recognizing and understanding how these multifarious elements interact with mitochondrial dysfunction is vital for formulating comprehensive treatment strategies to address this pressing health concern effectively (**Figure 2**).

### Regulation of mitochondrial biogenesis

Mitochondrial biogenesis represents a fundamental biological mechanism that guarantees the development and preservation of functional mitochondria, which are indispensable for energy metabolism, cellular signaling, and overall cellular viability (82). The modulation of mitochondrial biogenesis encompasses a sophisticated interaction among transcriptional co-activators, nuclear receptors, and transcription factors specific to mitochondria. Prominent signaling pathways include PGC-1α, NRF1, and TFAM as mentioned above. These signaling cascades are pivotal to mitochondrial biogenesis and substantially affect insulin sensitivity and metabolic well-being (83, 84).

Mitochondrial biogenesis is an essential biological process that determines the creation and sustenance of operational mitochondria, vital for energy metabolism, cellular signaling, and overall cellular survival (85). The mitochondrial biogenesis involves a complex interplay of transcriptional co-activators, nuclear receptors, and mitochondrial-specific transcription factors, thereby highlighting the intricate nature of this biological phenomenon (86).

**Figure 3:**
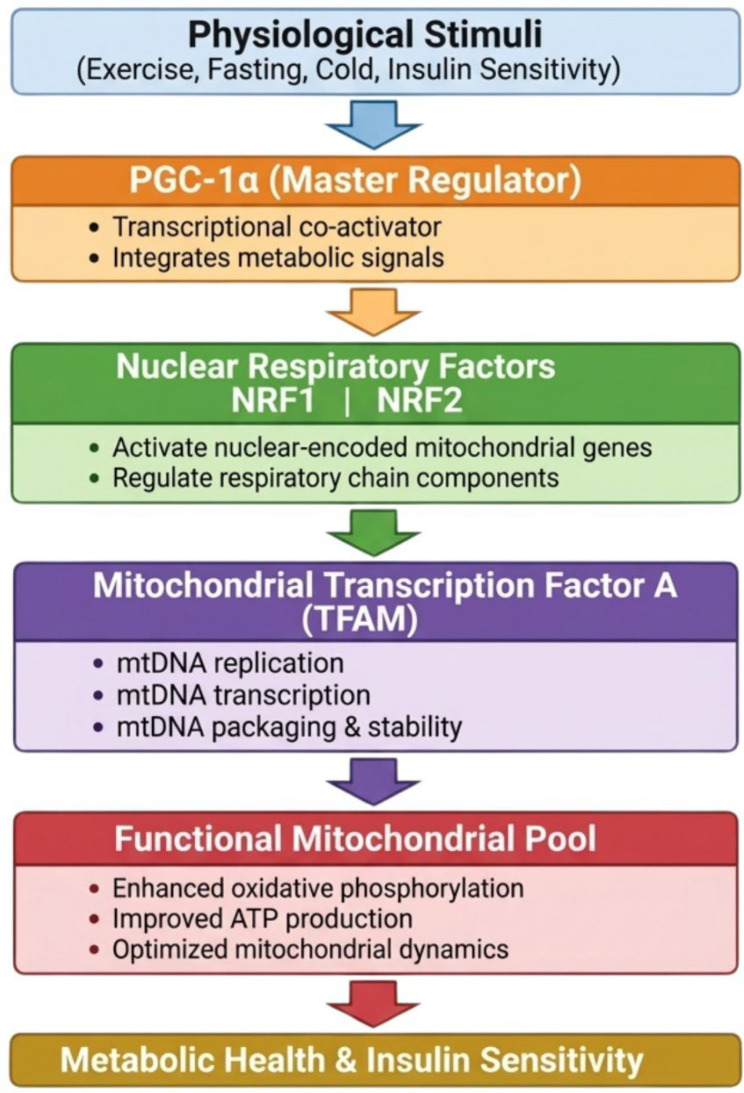
PGC-1α–mediated regulation of mitochondrial biogenesis. PGC-1α activates NRF1 and NRF2, leading to TFAM induction and increased mitochondrial DNA replication and transcription. At the same time, PGC-1α controls genes involved in oxidative phosphorylation and mitochondrial dynamics, promoting mitochondrial biogenesis, better metabolic health, and higher insulin sensitivity.PGC-1a, peroxisome proliferator-activated receptor γ co-activator 1α; NRF1, nuclear respiratory factor 1; NRF2, nuclear respiratory factor 2; TFAM, mitochondrial transcription factor A; mtDNA, mitochondrial DNA; ATP, adenosine triphosphate. Created in BioRender. Eldakhakhny, B (2026). https://BioRender.com/q6kgf7z.

To clarify the pathways regulating mitochondrial biogenesis, peroxisome proliferator-activated receptor γ co-activator-1α (PGC-1α) serves as a central regulatory hub (87). Activation of PGC-1α induces the expression of nuclear respiratory factors 1 and 2 (NRF1 and NRF2), which subsequently enhance transcription of mitochondrial transcription factor A (TFAM), a key protein required for mitochondrial DNA replication and transcription (88). Beyond this upstream role, PGC-1α also coordinates the expression of genes involved in oxidative phosphorylation and mitochondrial dynamics, thereby directly influencing mitochondrial efficiency and cellular energy production (89). Acting downstream of PGC-1α, NRF1 and NRF2 regulate multiple components of the mitochondrial respiratory chain, ensuring maintenance of mitochondrial integrity and function (90). TFAM, in turn, binds mitochondrial DNA to control its replication, transcription, and packaging, functioning as a critical downstream effector of both PGC-1α and NRF1 (91). Collectively, this tightly coordinated regulatory cascade highlights the pivotal role of mitochondrial biogenesis in metabolic health and insulin sensitivity and provides important mechanistic insight into the pathogenesis of metabolic disorders, as presented in **Figure 3**.

### Impact of mitochondrial dysfunction on insulin signaling pathways

The dysfunction within mitochondria causes an excessive production of reactive oxygen species (ROS), which substantially disrupts insulin signaling mechanisms by initiating various stress-sensitive pathways in conjunction with inflammatory responses that aggravate the condition (92). This cascade of events ultimately ends in modifications of the mitochondrial membrane potential and results in the release of cytochrome c. This process facilitates apoptosis and further exacerbates the damage inflicted upon the mitochondria, compounding the existing challenges associated with insulin resistance (93). The disruption of insulin signaling can be attributed to the accumulation of reactive oxygen species (ROS) and the associated inflammatory responses (6). These factors can hinder the insulin signaling pathways, especially in skeletal muscle, by negatively impacting the phosphorylation processes of insulin receptor substrates and other signaling molecules that are crucial for the proper functioning of insulin action (94).

Mitochondrial dysfunction in adipose tissue can significantly increase free fatty acids release, contributing to systemic insulin resistance and adversely impacting metabolic health (95). Similarly, impaired mitochondrial function in the liver disrupts the delicate balance of glucose homeostasis and insulin sensitivity, exacerbating hyperglycemia and complicating blood sugar regulation (96). Furthermore, dysfunction in skeletal muscle mitochondria is closely associated with the development of lipid-induced insulin resistance. This condition is characterized by a decrease in both mitochondrial content and functional capacity, resulting in a marked reduction in the muscle tissue’s ability to effectively uptake glucose (97).

Impaired mitochondrial function is a hallmark of insulin resistance, which is characterized by reduced mitochondrial biogenesis and oxidative phosphorylation alongside increased oxidative stress (98). These dysfunctions are particularly evident in skeletal muscle and liver tissues, contributing to the overall metabolic dysregulation observed in insulin-resistant states (59). Additionally, the interplay between ceramide levels and mitochondrial function is particularly interesting. Elevated ceramide levels within mitochondria can lead to coenzyme Q (CoQ) depletion, resulting in the loss of respiratory chain components and subsequent mitochondrial dysfunction (99). This ceramide-CoQ-respiratory chain nexus has been identified as a critical driver of insulin resistance, particularly in skeletal muscle, highlighting the complex relationship between lipid metabolism and mitochondrial health (93, 100).

Furthermore, mitochondrial remodeling plays a significant role in the pathogenesis of non-alcoholic fatty liver disease (NAFLD). This condition is characterized by notable changes in hepatic mitochondrial architecture, including increased activity of the tricarboxylic acid (TCA) cycle and activation of pyruvate dehydrogenase (PDH) (95, 101). These alterations are closely linked to insulin resistance and the accumulation of lipids in the liver, illustrating how mitochondrial dynamics can influence metabolic health. The interplay between mitochondrial function and metabolic disorders emphasizes the need for further research into strategies to enhance mitochondrial resilience as a potential therapeutic approach for conditions such as insulin resistance and NAFLD. Some of these examples are illustrated in **Table 4**.

**Table 4:** Key mechanisms, outcomes, of mitochondrial dysfunction and its relation to IR.

Mechanism/outcome	Description	Reference
Ceramide-CoQ-Respiratory Chain Nexus	Ceramide accumulation leads to CoQ depletion, impairing mitochondrial function.	(102, 103)
Oxidative Stress and ROS	Mitochondrial ROS impairs insulin signaling and promotes inflammation.	(80, 104)
Mitochondrial Dynamics and Biogenesis	Dysregulation of mitochondrial dynamics contributes to insulin resistance.	(4, 105, 106)
Genetic and Ethnic Factors	Specific genes and ethnic differences influence mitochondrial function.	(70, 107)
Insulin Resistance and T2DM	Mitochondrial dysfunction drives insulin resistance and T2DM progression.	(106, 108)
Metabolic Syndrome	Impaired mitochondrial biogenesis contributes to NAFLD and metabolic syndrome.	(105, 109)
Cardiovascular Complications	Mitochondrial dysfunction contributes to cardiac insulin resistance.	(70, 80)
Mitochondria-Targeted Antioxidants	MitoQ reduces oxidative stress and improves insulin sensitivity.	(104, 105)
Coenzyme Q Supplementation	CoQ supplementation restores mitochondrial function and insulin sensitivity.	(108, 110)
Redox-Active Compounds	Resveratrol and other compounds enhance mitochondrial biogenesis.	(111, 112)
Exercise and Metformin	Exercise and Metformin improve mitochondrial function and insulin sensitivity.	(113, 114)
Bioactive Food Components	Curcumin and astaxanthin mitigate insulin resistance.	(112)
Mitochondrial Transfer and Gene Therapy	Mitochondrial transfer restores metabolic health.	(114)

### Mitochondrial resilience and the pathogenesis of insulin resistance

Mitochondrial dysfunction is a central feature in insulin resistance (IR) pathogenesis (60, 98). Emerging research emphasizes on the concept of mitochondrial resilience to adapt to metabolic stress through mechanisms that preserve their function, integrity, and bioenergetic efficiency. Strengthening these adaptive responses has been shown to counteract the progression of insulin resistance and improve systemic metabolic outcomes (63, 115).

**Table 5** systematically outlines the key mechanisms by which mitochondrial resilience contributes to improved insulin sensitivity. It highlights each mechanism’s molecular mediators and signaling pathways, including mitochondrial biogenesis, dynamics, mitophagy, oxidative stress reduction, and anti-inflammatory responses. Additionally, **Table 5** describes the clinical implications associated with these mechanisms, such as improved glucose tolerance, reduced inflammation, and enhanced energy metabolism. Understanding these interconnected pathways offers valuable insights into therapeutic strategies that target mitochondrial function—such as exercise, pharmacological agents (e.g., Metformin, PPAR agonists), and dietary interventions (e.g., ketogenic diets). This integrated view underscores the potential of mitochondrial-targeted approaches in preventing and managing insulin resistance and related metabolic disorders.

**Table 5:** Mechanisms of mitochondrial resilience in counteracting insulin resistance: pathways, mediators, and clinical implications.

Mechanism of mitochondrial resilience	Key mediators & pathways	Effect on insulin sensitivity	Clinical implications	Reference
Enhanced Mitochondrial Biogenesis	PGC-1α, NRF1, TFAM, AMPK	Increases mitochondrial number and function, improving energy metabolism and insulin signaling (60)	Improved glucose tolerance; target in exercise, Metformin, and calorie restriction	(116, 117)
Improved Mitochondrial Dynamics (Fission/Fusion)	DRP1, MFN1/2, OPA1	Maintains mitochondrial quality and distribution, enhancing metabolic flexibility (63)	Reduces ectopic lipid accumulation; potential in neurodegenerative and metabolic diseases	(118, 119)
Mitophagy (Selective Mitochondrial Autophagy)	PINK1, Parkin, BNIP3, AMPK	Removes damaged mitochondria, reducing ROS and cellular stress	Enhances β-cell survival; promising in obesity and T2DM therapies	(120)
Reduction of Oxidative Stress	SOD2, Catalase, GPx, UCP2, Nrf2 pathway	Decreases mitochondrial ROS, preventing insulin signaling disruption	Protects pancreatic β-cells and insulin-responsive tissues	(121)
Improved ETC Efficiency & ATP Production	Complex I–V proteins, Coenzyme Q, ATP synthase	Enhances oxidative phosphorylation and glucose oxidation	Boosts muscle and liver insulin sensitivity; critical in endurance training outcomes	(11, 122)
Metabolic Substrate Flexibility	CPT1, PPARα, PDK4	Promotes fatty acid oxidation over glucose, decreasing lipotoxic intermediates	Mitigates insulin resistance in liver and muscle; relevant in ketogenic diets	(123, 124)
Anti-inflammatory Signaling	β-Hydroxybutyrate (βHB), SIRT3, IL-10, inhibition of NF-κB, NLRP3	Suppresses proinflammatory cytokines and inflammasome activation	Attenuates chronic inflammation-driven insulin resistance	(125–127)
Mitochondrial Uncoupling & Thermogenesis	UCP1, UCP2, UCP3, PGC-1α	Reduces ROS via mild uncoupling; increases energy expenditure	Helps in obesity prevention and insulin sensitization	(128–130)
Mitochondria-Nucleus Crosstalk (Retrograde Signaling)	ATF4, CHOP, Ca²^+^ signaling, ROS	Coordinates stress responses and gene expression involved in metabolism	Adaptation in chronic metabolic stress; relevant in mitochondrial-targeted therapies (131)	

### Enhancing mitochondrial resilience to combat insulin resistance

Non-pharmacological interventions offer accessible, sustainable approaches to promote mitochondrial health without relying on medications. Lifestyle modifications such as structured exercise, intermittent fasting, low-carbohydrate ketogenic diets, and targeted nutritional strategies can improve mitochondrial biogenesis, reduce oxidative stress, enhance mitophagy, and shift substrate utilization toward more efficient fat metabolism (132, 133). Emerging strategies like cold exposure and circadian rhythm optimization further support mitochondrial adaptability and insulin sensitivity (134, 135).

This enhancement is associated with improved insulin sensitivity through mechanisms that promote mitochondrial biogenesis while concurrently reducing oxidative stress levels. In addition to these lifestyle modifications, pharmacological strategies, including well-established medications like Metformin and dietary supplements that feature antioxidants and mitochondrial-targeted therapies such as mitoquinone, can effectively improve mitochondrial function and mitigate oxidative stress. These combined approaches contribute to a notable improvement in insulin sensitivity (9, 136).

In **Table 6**, we summarize the key non-pharmacological interventions that enhance mitochondrial resilience, detailing their mechanisms, molecular mediators, and clinical benefits. These interventions address the root metabolic disturbances underlying insulin resistance and align with broader preventive health measures. Integrating such strategies can significantly reduce the burden of metabolic diseases and improve long-term health outcomes.

**Table 6:** Non-pharmacological strategies to enhance mitochondrial resilience in insulin resistance.

Intervention	Mechanism of action	Key mediators/pathways	Impact on insulin sensitivity	Clinical implications	References
Aerobic & Resistance Exercise	Stimulates mitochondrial biogenesis, dynamics, and oxidative phosphorylatio	AMPK, PGC-1α, SIRT1, TFAM	Enhances glucose uptake, increases mitochondrial mass, reduces lipid intermediates	Improves insulin sensitivity in skeletal muscle and systemic glucose control	(137, 138)
Caloric Restriction (CR)/Intermittent Fasting (IF)	Promotes mitophagy and reduces oxidative damage	SIRT1, FOXO, AMPK, PINK1/Parkin	Enhances mitochondrial turnover and quality; reduces ROS and inflammatory cytokines	Improves metabolic flexibility and reduces IR risk in obesity and prediabetes	(139)
Low-Carbohydrate/Ketogenic Diet	Shifts energy metabolism to fat oxidation and ketone utilization	β-Hydroxybutyrate (βHB), PPARα, UCPs, Nrf2	Reduces glucose spikes and insulin demand, enhances mitochondrial respiration	Effective in reducing hepatic and peripheral insulin resistance; supports weight loss	(140–142)
High-Intensity Interval Training (HIIT)	Improves mitochondrial function and fusion/fission balance	DRP1, MFN2, PGC-1α	Enhances insulin signaling and mitochondrial metabolic flexibility	Time-efficient method to boost insulin sensitivity and cardiovascular health	(143, 144)
Cold Exposure/Thermogenesis Activation	Increases mitochondrial uncoupling and thermogenesis	UCP1, FGF21, PGC-1α	Promotes energy expenditure, reduces lipid accumulation	Potential non-dietary strategy to reverse metabolic inflexibility in IR	(145)
Nutraceutical-rich Diet (Polyphenols, Omega-3, etc.)	Provides antioxidants and enhances mitochondrial defenses	Nrf2, SIRT1, NF-κB inhibition	Reduces mitochondrial ROS and preserves mitochondrial integrity	Supports dietary management of IR; complements other lifestyle changes	(146)
Sleep Optimization	Reduces mitochondrial oxidative stress and supports repair	Melatonin signaling, circadian clock genes	Enhances mitochondrial function and hormone regulation	Protects against circadian misalignment-induced IR and metabolic syndrome	(147, 148)

The role of targeted interventions in ameliorating insulin resistance is complex and multifaceted, encompassing a diverse array of dietary, pharmacological, and lifestyle strategies that synergistically enhance insulin sensitivity and mitochondrial functionality (20). Among the most prominent methodologies is the adoption of low-carbohydrate ketogenic diets, which are characterized by elevated fat consumption, moderate protein intake, and a significant reduction in carbohydrate consumption (149). This dietary paradigm instigates a profound metabolic shift, converting the body’s primary energy substrate from glucose to the oxidation of ketone bodies. This transition has been empirically shown to improve insulin sensitivity markedly (150).

The pathways that highlight these enhancements are varied and intricate. A pivotal mechanism involves attenuating hepatic glucose output, achieved through inhibiting gluconeogenesis, thereby effectively lowering blood glucose concentrations. Furthermore, a ketogenic dietary regimen promotes fatty acid oxidation, which mitigates ectopic lipid accumulation within skeletal muscle and hepatic tissues (151). Reducing lipid storage is critical, as excessive fat deposition has been identified as a significant contributor to insulin resistance. Additionally, ketogenic diets have been correlated with increased mitochondrial biogenesis, as evidenced by elevated expression levels of PGC-1α and associated factors, leading to improvements in mitochondrial efficacy and functionality (152, 153).

Weight management, accomplished through dietary modifications and lifestyle interventions, is critical for enhancing insulin sensitivity. Such approaches promote mitochondrial biogenesis and mitigate lipid accumulation within skeletal muscle and hepatic tissues (133). Empirical evidence indicates that weight loss elevates the expression of mitochondrial biogenesis-related factors, such as PGC-1α, while concurrently decreasing ectopic lipid deposition. This optimization of mitochondrial dynamics, encompassing mitochondrial fusion and fission processes, is indispensable for maintaining effective mitochondrial function (154, 155).

While lifestyle and nutritional strategies play a foundational role in enhancing mitochondrial resilience, pharmacological interventions provide additional or alternative means to target mitochondrial dysfunction in IR. These agents act on various molecular pathways to restore mitochondrial bioenergetics, reduce oxidative stress, enhance mitophagy, and support metabolic flexibility, ultimately improving insulin sensitivity across tissues (156).

The table below (**Table 7**) outlines key potential pharmacological interventions (conventional and emerging) that enhance mitochondrial resilience in the context of IR. Each drug or compound is associated with specific mechanisms of action and signaling pathways that contribute to improved mitochondrial health. For example, Metformin, a first-line therapy for type 2 diabetes mellitus (T2DM), activates AMPK and modulates mitochondrial complex I activity, improving glucose metabolism and reducing hepatic insulin resistance. Similarly, Thiazolidinediones activate PPARγ to improve adipocyte mitochondrial function, while GLP-1 receptor agonists stimulate mitochondrial biogenesis and reduce inflammation. Other agents, including mitochondria-targeted antioxidants, SIRT1 activators, and nutraceuticals like berberine and Coenzyme Q10, act through mitochondrial pathways that support oxidative balance and metabolic regulation.

**Table 7:** Pharmacological strategies to enhance mitochondrial resilience in insulin resistance.

Pharmacological agent	Mechanism of action	Key mediators/pathways	Impact on insulin sensitivity	Clinical implications	Reference
Metformin	Activates mitochondrial AMPK signaling and inhibits complex I of ETC	AMPK, ACC, IRS1/2, GLUT4	Increases glucose uptake, reduces hepatic gluconeogenesis, improves mitochondrial function	First-line therapy in T2DM; reduces insulin resistance and inflammation	(157, 158)
Thiazolidinediones (e.g., Pioglitazone)	Activates PPARγ and improves mitochondrial biogenesis and lipid metabolism	PPARγ, PGC-1α, Adiponectin	Enhances adipocyte insulin sensitivity and reduces ectopic fat	Used in T2DM and NAFLD; improves lipid profile and IR	(159, 160)
Mitochondria-targeted Antioxidants (e.g., MitoQ, SkQ1)	Scavenge mitochondrial ROS and protect mitochondrial membrane potential	ETC complexes, CoQ10 analogs	Prevents oxidative damage, preserves mitochondrial integrity	Experimental; potential therapy for metabolic and neurodegenerative conditions	(161, 162)
SIRT1 Activators (e.g., Resveratrol, SRT2104)	Mimic caloric restriction by activating mitochondrial biogenesis and autophagy	SIRT1, PGC-1α, FOXO	Improves mitochondrial quality control and oxidative metabolism	Investigational use; promising in age-related metabolic decline and IR	(123, 124, 163, 164)
GLP-1 Receptor Agonists (e.g., Liraglutide)	Enhances mitochondrial biogenesis and reduces inflammation	GLP-1R, cAMP, AMPK, PKA	Improves insulin signaling and β-cell survival	Used in T2DM and obesity; offers cardiovascular and hepatic benefits	(165, 166)
Berberine	Activates AMPK and improves mitochondrial dynamics	AMPK, SIRT3, Drp1/MFN2	Increases fatty acid oxidation and reduces ER stress	Botanical compound with insulin-sensitizing effects similar to Metformin	(167)
Coenzyme Q10	Supports ETC activity and reduces oxidative stress	Complex I–III, ATP synthase	Enhances mitochondrial efficiency and reduces ROS	Adjunct supplement in T2DM and statin-induced myopathy	(165, 168)
Carnitine Supplements (e.g., L-carnitine)	Facilitates mitochondrial fatty acid transport and oxidation	CPT1, PPARα	Reduces lipid-induced insulin resistance	Supportive role in fatty acid metabolism disorders and metabolic syndrome	(169)

## Discussion

**Figure 4:**
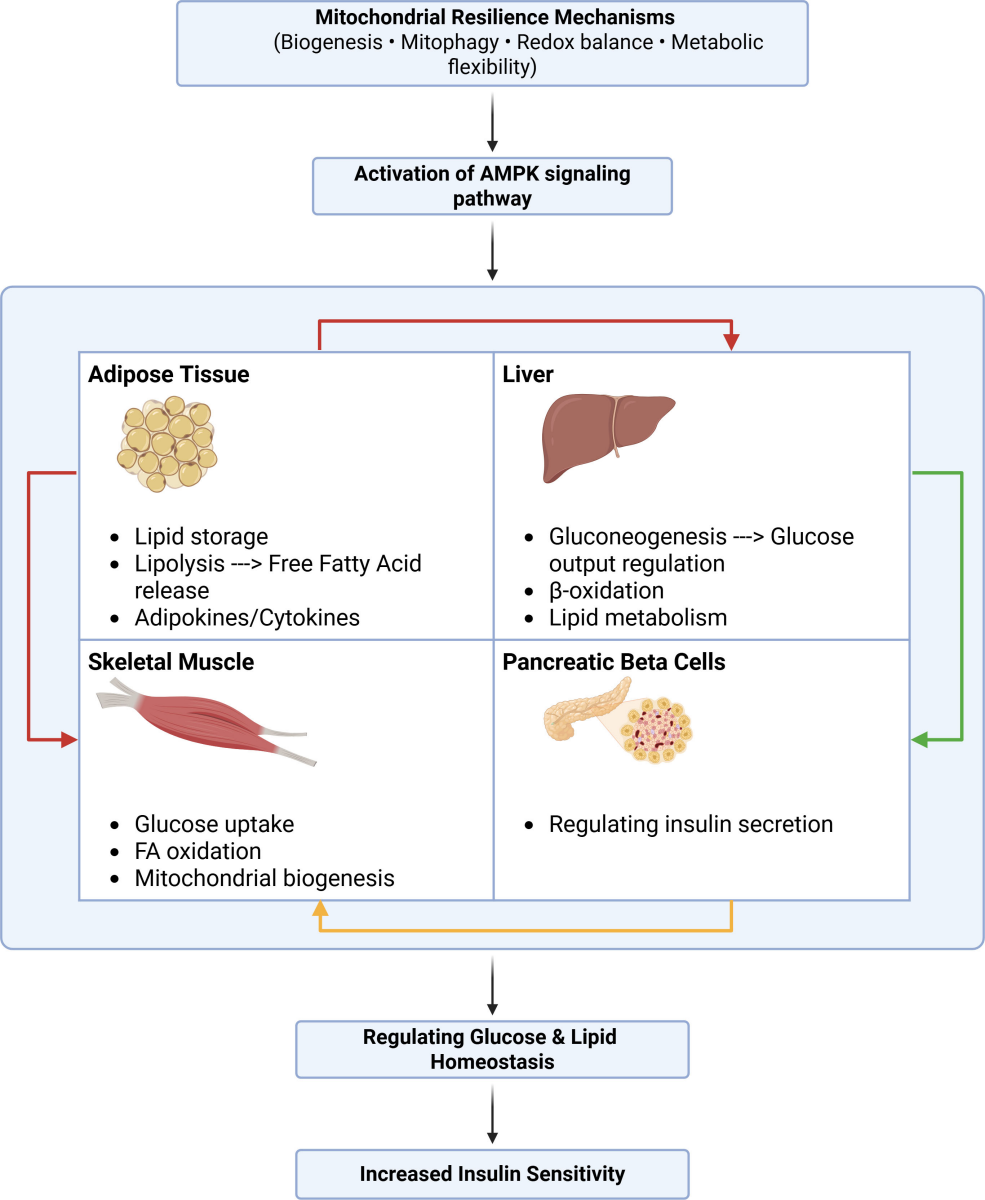
Flow-chart representation of mitochondrial resilience pathways and their tissue-specific interactions in regulating systemic insulin resistance. Mitochondrial adaptive mechanisms, including biogenesis, mitophagy, redox regulation, and metabolic flexibility, operate across key metabolic tissues such as skeletal muscle, liver, adipose tissue, and pancreatic β-cells. Through metabolic substrate exchange and endocrine signaling (e.g., adipokines, cytokines, and free fatty acids), these tissues communicate to influence whole-body glucose and lipid homeostasis, thereby modulating insulin sensitivity. Created in BioRender. Eldakhakhny, B. (2026). https://BioRender.com/q7x4hau.

The findings of this scoping review underscore the critical role of mitochondrial resilience in restoring insulin sensitivity and mitigating metabolic dysfunction. To integrate these tissue-specific mechanisms, we propose a conceptual model illustrating cross-tissue mitochondrial resilience pathways regulating systemic Insulin Resistance (**Figure 4**).

Enhancing mitochondrial function through non-pharmacological (e.g., exercise, ketogenic diets, fasting) and pharmacological strategies (e.g., Metformin, GLP-1 agonists) provides a dual-pronged approach to managing insulin resistance. These interventions improve mitochondrial biogenesis, dynamics, and redox balance and reduce chronic inflammation and lipotoxicity, which are the central contributors to IR. From a practical standpoint, integrating lifestyle modifications with targeted therapeutics may offer synergistic benefits in patient management, especially in individuals at high metabolic risk. These insights can inform clinical decision-making, guide patient education, and inspire the development of personalized treatment protocols.

Integrating mitochondrial resilience into managing IR offers significant promise for advancing precision and personalized medicine. Precision medicine aims to tailor healthcare strategies based on individual variability in genes, environment, and lifestyle (170, 171). Since mitochondrial function is highly influenced by genetic background, metabolic state, diet, and physical activity, understanding the mechanisms of mitochondrial resilience provides a molecular basis for patient-specific interventions.

One of the key insights from this review is the identification of diverse pathways, such as AMPK activation, PGC-1α-mediated mitochondrial biogenesis, and mitophagy regulation, that can be selectively targeted depending on an individual’s metabolic profile (172, 173). For example, patients with a genetic predisposition to impaired mitochondrial dynamics (e.g., MFN2 mutations) may benefit more from interventions that enhance mitochondrial fusion or prevent fragmentation (174, 175). Similarly, individuals with elevated oxidative stress or inflammation may respond more effectively to antioxidant-based therapies or anti-inflammatory dietary strategies, such as ketogenic or fasting-mimicking regimens (176–178).

The link between mitochondrial dysfunction and insulin resistance (IR) is intricate and may vary depending on tissue, metabolic state, and disease progression. While mitochondrial dysfunction is often viewed as a cause of IR, evidence indicates a more complex, possibly reciprocal relationship. Reduced mitochondrial oxidative capacity can cause lipid intermediate buildup, increased oxidative stress, and energy imbalance in cells, impairing insulin signaling and leading to metabolic inflexibility (179). Conversely, chronic nutrient excess and faulty insulin signaling can induce secondary mitochondrial changes, like decreased biogenesis, altered dynamics, and reduced oxidative capacity (60). This two-way relationship implies that mitochondrial dysfunction both contributes to and results from metabolic disturbances, shaped by the specific physiological or pathological setting (5, 180, 181). Understanding this relationship is essential for interpreting research and developing therapies to improve mitochondrial health in metabolic diseases.

From a clinical perspective, the identification and application of mitochondrial resilience biomarkers may provide valuable opportunities for the early detection and management of insulin resistance (60). Biomarkers representing key components of mitochondrial resilience, including mitochondrial biogenesis (e.g., PGC-1α and TFAM), mitochondrial dynamics (e.g., MFN1/2 and DRP1), mitophagy pathways (e.g., PINK1 and Parkin), and redox regulation (e.g., NRF2-regulated antioxidant enzymes and circulating oxidative stress markers) may serve as indicators of mitochondrial functional integrity and metabolic flexibility (182). These biomarkers could potentially be assessed through circulating metabolites, gene expression signatures, or protein markers obtained from blood or tissue samples. This perspective aligns with the growing recognition of mitochondrial health as a central factor in metabolic disorders, particularly in understanding the mechanisms underlying insulin resistance and its implications for diabetes management (20).

Integrating mitochondrial resilience biomarkers into clinical practice may facilitate the early identification of individuals with reduced mitochondrial adaptive capacity before overt metabolic dysfunction develops (183). In addition, such markers may help stratify patients according to their metabolic phenotype and support personalized interventions, including tailored nutritional strategies, structured exercise programs, and pharmacological therapies targeting mitochondrial function (184). Furthermore, longitudinal monitoring of these biomarkers could assist in evaluating therapeutic responses and tracking disease progression, thereby strengthening the translational connection between mitochondrial biology and precision metabolic medicine (185). Ultimately, this approach may contribute to the development of targeted therapeutic strategies and improved clinical outcomes in metabolic disorders.

Moreover, non-pharmacological interventions such as exercise, caloric restriction, or thermogenic stimulation can be optimized based on individual mitochondrial capacity and metabolic flexibility (145, 186). Personalized exercise prescriptions, guided by mitochondrial function biomarkers (e.g., VO_2_ max, mitochondrial DNA copy number, lactate threshold), may enhance intervention efficacy and adherence (187, 188). Nutrigenomic testing could further inform dietary modifications that support mitochondrial health and insulin sensitivity, allowing for targeted nutritional interventions based on a patient’s genetic susceptibility to IR or poor fat oxidation (4).

In pharmacological terms, agents such as metformin, GLP-1 receptor agonists, mitochondrial-targeted therapies for insulin resistance, and mitochondrial-directed antioxidants have been proposed or investigated as treatments that may be rationally selected based on underlying molecular and mitochondrial mechanisms contributing to insulin resistance (189–191). Biomarkers such as circulating β-hydroxybutyrate levels, mitochondrial reactive oxygen species, and transcriptomic profiles related to energy metabolism have been proposed as tools to monitor treatment responses in insulin resistance and diabetes and may support adaptive or personalized therapeutic strategies (192–194).

Ultimately, the findings of this scoping review support a shift from a one-size-fits-all approach toward stratified care models, where therapeutic strategies are aligned with mitochondrial phenotypes and individual pathophysiological features (195–197). As we continue to unravel the complexities of mitochondrial biology in insulin resistance, this knowledge will become instrumental in designing personalized protocols for prevention, early intervention, and long-term management of metabolic diseases.

Mitochondrial resilience plays a critical role in metabolic flexibility and insulin sensitivity, yet findings in this area remain inconsistent. Ketogenic or very low-carbohydrate diets have been reported in some studies to enhance mitochondrial efficiency and promote fatty acid oxidation (198–200). However, other research indicates variable effects on insulin sensitivity and mitochondrial adaptations, influenced by factors such as diet duration, macronutrient composition, and baseline metabolic status (201, 202). These discrepancies underscore the importance of personalized dietary strategies that account for individual metabolic profiles to optimize mitochondrial function and improve insulin sensitivity. Future research should focus on identifying metabolic markers capable of predicting individual responses to dietary interventions, enabling tailored approaches to support mitochondrial health.

Similarly, exercise is a well-established stimulus for mitochondrial biogenesis and metabolic enhancement, though the extent and nature of these adaptations vary across individuals and tissues (203, 204). Variations in training intensity, duration, age, metabolic health, and genetic background likely contribute to this heterogeneity (205). This variability highlights the complex regulation of mitochondria and suggests that mitochondrial resilience arises from an interplay between lifestyle, metabolic context, and intrinsic cellular factors (206, 207). Consequently, personalized exercise regimens may be necessary to maximize mitochondrial adaptations and overall metabolic health.

This review has several limitations to keep in mind when interpreting the results. The literature included encompasses different experimental designs, populations, and methods, leading to variability that may influence the consistency of reported outcomes. Much of the understanding of mitochondrial resilience and its link to insulin resistance is derived from cell culture and animal studies, which might not fully reflect the complexity of human metabolism. Another limitation is the small number of well-designed, long-term clinical trials focused on mitochondrial pathways for managing insulin resistance and related metabolic conditions. Additionally, as a scoping review, the purpose was to outline existing evidence rather than conduct a detailed critical analysis or provide a quantitative synthesis. While this approach offers a broad perspective, it may reduce the depth of methodological assessment for the studies included.

### Recommendation

Given the multifactorial nature of insulin resistance and the growing recognition of mitochondrial dysfunction as a central contributor, we recommend a paradigm shift in research and clinical practice toward precision and personalized medicine approaches. Future studies should aim to stratify patients based on individual mitochondrial phenotypes, genetic polymorphisms, metabolic profiles, and lifestyle factors to identify better who will benefit most from specific interventions targeting mitochondrial resilience.

Research should focus on developing and validating mitochondrial health biomarkers, such as mitochondrial DNA copy number, PGC-1α expression, oxidative stress markers, or mitochondrial respiration capacity. These could serve as diagnostic tools and treatment monitoring indicators. Furthermore, integrating multi-omics technologies (genomics, transcriptomics, metabolomics, and epigenomics) into clinical trials can help elucidate patient-specific mitochondrial dysfunction pathways and tailor interventions accordingly.

In clinical settings, personalized intervention plans should be formulated based on a patient’s metabolic flexibility, physical activity capacity, dietary habits, and genetic risk factors. For example, patients with poor mitochondrial fatty acid oxidation may benefit more from a carbohydrate-restricted ketogenic diet. In contrast, others with high inflammatory profiles may respond better to fasting protocols or antioxidant-based therapies. Pharmacologic strategies, such as Metformin, GLP-1 receptor agonists, or SIRT1 activators, should be selected and dosed according to individual molecular signatures and predicted drug response.

To operationalize this approach, interdisciplinary collaborations between clinicians, molecular biologists, nutritionists, and bioinformaticians are essential. Research funding should prioritize longitudinal cohort studies integrating lifestyle, molecular, and pharmacological data to assess the long-term benefits of personalized mitochondrial-targeted interventions on insulin resistance and its complications.

So, advancing the field of mitochondrial medicine in insulin resistance requires both mechanistic studies and translational frameworks that embrace individual variability. Tailoring interventions to mitochondrial function improves therapeutic efficacy and aligns with the broader goals of reducing healthcare burden and enhancing patient-centered outcomes in metabolic disease management.

## Conclusion

This scoping review highlights the pivotal role of mitochondrial resilience in the pathogenesis and potential reversal of insulin resistance. By mapping the current evidence on non-pharmacological and pharmacological interventions, we demonstrate how enhancing mitochondrial biogenesis, dynamics, oxidative balance, and quality control mechanisms can significantly improve insulin signaling and metabolic health. These findings reinforce the concept that mitochondria are not merely energy producers but are central regulators of cellular homeostasis, inflammation, and nutrient sensing in insulin-sensitive tissues.

Importantly, this review supports a growing movement toward precision and personalized medicine in managing metabolic disorders. As mitochondrial function varies greatly between individuals due to genetic, environmental, and lifestyle factors, tailoring interventions to mitochondrial profiles offers a promising strategy for individualized care. Whether through personalized exercise regimens, targeted dietary interventions, or mitochondria-specific pharmacological agents, enhancing mitochondrial resilience can serve as a strategic entry point for treating insulin resistance in a patient-specific manner.

Finally, targeting mitochondrial health offers a scientifically grounded and clinically relevant framework for advancing metabolic care. Future research that integrates biomarker discovery, patient stratification, and individualized therapy design will be crucial in translating these mechanistic insights into effective, personalized treatments for insulin resistance and related disorders.
